# Fabry Disease: Cardiac Implications and Molecular Mechanisms

**DOI:** 10.1007/s11897-024-00645-1

**Published:** 2024-01-30

**Authors:** David Weissman, Jan Dudek, Vasco Sequeira, Christoph Maack

**Affiliations:** Department of Translational Research, Comprehensive Heart Failure Center, University Hospital Würzburg, Am Schwarzenberg 15, Haus A15, 97078 Würzburg, Germany

**Keywords:** Fabry disease, Fabry cardiomyopathy, Metabolic dysfunction, Oxidative stress, Inflammation, Fibrosis

## Abstract

**Purpose of Review:**

This review explores the interplay among metabolic dysfunction, oxidative stress, inflammation, and fibrosis in Fabry disease, focusing on their potential implications for cardiac involvement. We aim to discuss the biochemical processes that operate in parallel to sphingolipid accumulation and contribute to disease pathogenesis, emphasizing the importance of a comprehensive understanding of these processes.

**Recent Findings:**

Beyond sphingolipid accumulation, emerging studies have revealed that mitochondrial dysfunction, oxidative stress, and chronic inflammation could be significant contributors to Fabry disease and cardiac involvement. These factors promote cardiac remodeling and fibrosis and may predispose Fabry patients to conduction disturbances, ventricular arrhythmias, and heart failure. While current treatments, such as enzyme replacement therapy and pharmacological chaperones, address disease progression and symptoms, their effectiveness is limited.

**Summary:**

Our review uncovers the potential relationships among metabolic disturbances, oxidative stress, inflammation, and fibrosis in Fabry disease–related cardiac complications. Current findings suggest that beyond sphingolipid accumulation, other mechanisms may significantly contribute to disease pathogenesis. This prompts the exploration of innovative therapeutic strategies and underscores the importance of a holistic approach to understanding and managing Fabry disease.

## Introduction

Fabry disease (FD) is an X-linked, inherited metabolic condition classified as a lysosomal storage disorder (LSD). It is caused by mutations in the GLA gene, which encodes the lysosomal enzyme α-galactosidase A (α-Gal A) [1]. This enzyme is crucial for the lysosomal degradation of glycosphingolipids, particularly globotriaosylceramide (Gb3). Gb3 is a key cell membrane component involved in signaling transduction and cell-to-cell interactions [2]. Furthermore, it serves as a receptor for specific bacterial toxins [3]. In FD, reduced or absent α-Gal A activity results in the accumulation of Gb3 and its deacylated form, globotriaosylsphingosine (lyso-Gb3), in various tissues and organs. FD manifests systemically, affecting the gastrointestinal tract, kidney, and central nervous system. Early manifestations include pain crises, acroparesthesias, angiokeratoma, hypohidrosis, and cornea verticillate [4]. However, cardiovascular complications, primarily involving the heart, represent the leading cause of death in Fabry patients [5].

Fabry cardiomyopathy manifests typically as left ventricular hypertrophy (LVH), myocardial fibrosis, arrhythmias, and heart failure (HF) [4, 5]. Over 1000 GLA gene mutations are reported, categorized into classic and late-onset variants [6]. The classic variant presents early in life with severe symptoms and correlates with an α-Gal A activity below 1%. In contrast, the late-onset variant manifests later, primarily affecting the heart, and is associated with residual α-Gal A activity [7]. Although the prevalence has traditionally been estimated at 1 in 20,000 to 60,000, newborn screenings suggest that FD prevalence might be as frequent as 1 in 8800 [8, 9].

The cornerstone of FD management is to halt disease progression and prevent irreversible organ damage. Disease-specific therapies such as enzyme replacement therapy (ERT) and the pharmacological chaperone migalastat play pivotal roles [10, 11]. ERT, which aims at replacing or restoring deficient enzyme activity, has significantly advanced FD management, ameliorating a variety of symptoms and improving life expectancy [12, 13]. Migalastat, an oral chaperone, has been identified as particularly beneficial for FD patients with amenable mutations that are associated with low residual α-Gal A activity [14, 15]. Additionally, substrate reduction therapy introduces a novel approach to managing FD by specifically targeting glycosphingolipid biosynthesis, thus reducing their accumulation in tissues [16]. Comprehensive clinical monitoring is also crucial, with innovations like multiparametric scoring systems and advanced imaging offering deeper insights into disease progression and treatment efficacy [17].

While current therapies provide significant benefits in addressing FD-associated complications, they come with inherent limitations, which include immunogenicity, high costs, and inconsistent effectiveness in halting disease progression, especially in advanced disease stages and for cardiac involvements [18]. This underscores the need for a deeper understanding of the ancillary disease mechanisms at play, extending beyond sphingolipid accumulation. An emerging area of interest is the interplay between metabolic dysfunction and oxidative stress, both closely linked to inflammation and fibrosis. In this review, we delve into experimental and clinical evidence on these mechanisms, underscoring the significance of ongoing research.

## Fabry Cardiomyopathy: Clinical Perspective and Pathogenic Mechanisms

Fabry cardiomyopathy is characterized by LVH and diastolic dysfunction, with systolic function typically preserved in most patients. Diastolic dysfunction can manifest early in FD, even preceding hypertrophy onset [19, 20•]. The incidence of LVH tends to increase with aging, manifesting more commonly in male patients, and correlating with diminished α-Gal A activity. The presentation of LVH in FD patients is diverse, encompassing septal, concentric, and eccentric to apical hypertrophy [21]. Although LVH is prevalent in both classical and late-onset variants, the LV mass index is distinctly elevated in the classical variant. This differential presentation highlights the complex nature of the disease and the need for comprehensive patient assessment [22].

Recent studies demonstrate that Gb3 accumulation affects several cell types in the heart, including cardiomyocytes, endothelial and smooth muscle cells, but also immune cells, and fibroblasts [23–26, 27•]. Sphingolipid deposits within cardiomyocytes have been associated with myofilament degradation and dysfunction [28•]. This accumulation contributes to the onset of LVH and fibrosis, which ultimately leads to cardiac dysfunction [29]. They also impact ion homeostasis, especially sodium (Na^+^) and calcium (Ca^2+^) channel functions, resulting in hypertrophic gene expression and impaired conduction in both ventricular and atrial myocardium, manifesting as electrocardiographic (ECG) changes comprising bradycardia, PQ interval shortening, and/or atrioventricular, but also an increased risk for atrial or ventricular tachycardia or fibrillation [30, 31].

Sphingolipid accumulation in the hearts of Fabry patients is believed to trigger immune cell activation and infiltration [32]. This response is influenced by both Gb3 and lyso-Gb3, which act as antigens, and by the activation of the toll-like receptor-4 (TLR4) signaling pathway [33]. This activation results in local inflammation and pro-fibrotic signaling [33, 34]. Cross-talk between immune cells and fibroblasts may also play a crucial role in promoting myofibroblast differentiation and subsequent extracellular matrix (ECM) deposition [35]. These alterations not only contribute to cardiac fibrosis and stiffness, but also lead to conduction abnormalities, resulting in arrhythmias that can manifest as palpitations and syncope [30, 36]. Additionally, they contribute to the development of LVH and vascular involvement that give rise to anginal pain [30, 36, 37].

Sphingolipid accumulation in cardiac endothelial and smooth muscle cells is associated with endothelial dysfunction and small vessel ischemia. This is supported by the prevalent incidence of myocardial infarction, particularly in patients with LVH, despite the absence of obstructive coronary artery disease [38, 39]. Beyond the mechanical and functional implications of sphingolipid deposition, emerging evidence suggests that alterations in energy metabolism, particularly altered mitochondrial function and imbalances in energy production, may play a role in the pathogenesis of FD [31, 40•]. The ensuing section will further explore this association between metabolic disturbances and clinical manifestations.

## Mitochondrial Dysfunction in Fabry Disease: Cardiac Implications

Mitochondrial dysfunction, particularly imbalances in energy molecules such as phosphocreatine (PCr), adenosine diphosphate (ADP), and adenosine triphosphate (ATP), along with alterations in mitochondrial structure and respiratory function, has emerged as an important contributor to the pathogenesis of different LSDs, including FD [41, 42]. Using phosphorus-31 magnetic resonance spectroscopy (^31^P-MRS), a non-invasive technique that can assess in vivo cellular bioenergetics, an association between reduced PCr/ATP ratio and the progression of LV mass has been observed in the heart of FD patients [43•]. A subsequent in vivo study supported these findings, showing further reduced PCr and ATP concentrations in the heart of FD patients [40•].

Similar bioenergetic disturbances have been documented in other LSDs. For instance, neurons from a mouse model of Gaucher disease (GD), an LSD caused by mutations in the GBA1 gene leading to an accumulation of glucocerebrosides [44]**,** showed a reduced ATP/ADP ratio. This reduction was associated with a disturbed mitochondrial membrane potential and reduced mitochondrial Ca^2+^ uptake [45]. Parallel observations were made in an in vitro model of Pompe disease (PD), another LSD caused by a deficiency of acid α-glucosidase enzyme activity and glycogen accumulation [46]. Using induced pluripotent stem cells (iPSCs)–derived myocytes from PD patients, Yoshida et al. [47] reported a reduced adenylate energy charge, a ratio representing the cell’s energy status defined as (ATP + ADP / 2) / (ATP + ADP + AMP). Furthermore, a decline in the phosphocreatine to creatine (PCr/Cr) ratio and nicotinamide adenine dinucleotide (NAD^+^)/nicotinamide adenine dinucleotide hydride (NADH) ratio was observed, both observations indicative of an impaired cellular energy metabolism and diminished mitochondrial oxidative function. The shared bioenergetic disturbances across various LSDs underscore the significance of mitochondrial dysfunction and energetic as a potential underlying mechanism in these disorders, warranting further research and therapeutic attention.

Despite some reports of a compensatory increase in mitochondrial respiration in immortalized cell models of FD [33], Lücke et al. [48] demonstrated reduced activities of key mitochondrial respiratory chain enzymes (i.e., complexes I, IV, and V), accompanied by decreased levels of phosphorus nucleotides, in skin fibroblasts from FD patients. Ivanova et al. [49] also identified compromised mitochondrial function, accompanied by impaired autophagy and accumulation of damaged and/or long-lived mitochondria in peripheral blood mononuclear cells from FD and GD patients. These findings suggest that in the broader context of LSDs, lysosomal dysfunction can influence mitochondrial homeostasis, leading to cellular metabolic dysregulation.

In line with these observations, Schumann et al. [50] documented impaired autophagy function, accompanied by fragmented mitochondria with disrupted cristae, in renal cells from FD patients. Elevated mitochondrial oxidative phosphorylation and accumulation of tricarboxylic acid cycle (TCA) intermediates were also documented, suggesting a compensatory mechanism to meet elevated energy demands in these cells. Notably, Sirtuin 1, a key metabolic sensor, was found upregulated, pointing to the activation of metabolic adaptive stress responses [51]. These insights highlight the interplay between lysosomal anomalies in FD and mitochondrial function, suggesting that mitochondrial dysfunction might not only contribute to cardiac involvement, but could also underlie metabolic dysfunction and associated functional impairments in various cell lines and tissues of FD patients.

Chou et al. [52•] reported a reduced mitochondrial fatty acid oxidation and a pathological shift towards glycolysis-dominant metabolism for ATP production in iPSCs-derived cardiomyocytes (iPSC-CMs) from FD patients. These alterations were associated with a downregulation of key enzymes involved in fatty acid transport, including mitochondrial carnitine palmitoyltransferase 1 (CPT1) and 2 (CPT2). These changes were accompanied by an upregulation of cardiac hypertrophy–related genes and impaired contractility. Notably, metabolic disturbance persisted after ERT treatment, despite a reduction in Gb3 deposits, reinforcing the idea that FD pathogenesis extends beyond mere sphingolipid accumulation, while emphasizing the need for complementing ERT with alternative therapeutic strategies.

Birket et al. [31] also exanimated iPSC-CMs from FD patients, identifying an upregulation of the lysosomal integral membrane protein-2 (LIMP-2). LIMP-2 is a trafficking receptor for the β-glucocerebrosidase, a lysosomal enzyme involved in sphingolipid degradation [53]. It can be speculated that the upregulation of this protein not only serves as a compensatory mechanism to support Gb3-degrading enzymes in the lysosomes but may also modulate metabolism indirectly. In fact, LIMP-2 was recently identified as a modulator of mitochondrial oxidative phosphorylation through the mechanistic target of the rapamycin complex 1 pathway [54]. Furthermore, the authors found a downregulation of the mitochondrial endonuclease G, an important regulator of mitochondrial function and lipid metabolism, involved in maladaptive cardiac hypertrophy [55]. In light of this evidence, it becomes imperative to delve deeper into the role of mitochondrial dysfunction in FD, exploring potential strategies that target metabolic dysfunction as a potential therapeutic approach.

## Oxidative Stress in Fabry Disease

Oxidative stress is characterized by an imbalance between the production of reactive oxygen species (ROS), primarily during mitochondrial oxidative phosphorylation and other enzymatic reactions, and their detoxification by the antioxidant system [56]. This imbalance plays a crucial role in various pathological conditions and is also believed to play a significant role in the pathogenesis of FD. For example, Chen et al. [57] documented increased plasma levels of 8-hydroxydeoxyguanosine (8-OHdG), a biomarker for oxidative DNA damage [58], in patients with Fabry cardiomyopathy. This was further corroborated by Simoncini et al. [59], who identified elevated oxidative stress markers in FD patients, including advanced oxidation protein products, and a reduction in antioxidant defenses, such as ferric reducing antioxidant power and thiol groups. Notably, these markers were found to be altered in untreated patients showing early signs of organ damage, despite normal plasma lyso-Gb3 levels [59]. This evidence emphasizes the role of oxidative stress in FD, suggesting also the potential of oxidative stress markers for early detection.

Chimenti et al. [60•] also reported increased 8-OHdG, accompanied by increased Gb3 accumulation and apoptosis, in cardiomyocytes from FD patients. These findings were associated with an upregulation of inducible nitric oxide synthase (iNOS) and increased nitrotyrosine levels, both indicative of oxidative and nitrosative stress. A complementary study by Shen et al. [61] further identified a deficiency in tetrahydrobiopterin (BH4), an essential cofactor for NOS, in heart and kidney biopsies from FD patients. Oxidized or diminished BH4 imposes the risk of “NO uncoupling,” a process where NOS produces superoxide anion besides nitric oxides [62]. The study also observed an inverse correlation between Gb3 levels and BH4 levels, linking BH4 deficiency to oxidative stress through a reduced antioxidant capacity and NOS uncoupling. Notably, BH4 deficiency was not corrected by ERT, further underscoring the limitations of current treatment options in addressing ancillary biochemical disturbances beyond sphingolipid accumulation [61]. Interestingly, glutathione (GSH), a key component in antioxidant defense [63], was found downregulated in male Fabry mouse models, compared to females, emphasizing the role of sex-specific differences in the pathogenesis of FD.

Tseng et al. [64] found that Gb3 accumulation in FD-iPSC-derived vascular endothelial cells suppressed superoxide dismutase 2 (SOD2) expression, an important mitochondrial antioxidant, leading to mitochondrial ROS production and vascular dysfunction. These changes were accompanied by increased AMP-activated protein kinase (AMPK) activity, a regulator of cellular energy homeostasis [65], suggestive of metabolic imbalances. Kim et al. [66] observed a similar increase in mitochondrial ROS in FD-iPSC-derived kidney organoids, accompanied by mitochondrial dysfunction. A corresponding decrease in GSH levels further supported the association with oxidative stress. Notably, these alterations could be attenuated with GSH treatment. In a series of studies, Biancini et al. [67–69] further highlighted disturbances in GSH metabolism in FD patients, accompanied by increased lipid peroxidation and nitric oxide levels. These changes were correlated to elevated plasma levels of pro-inflammatory cytokines, highlighting the role of oxidative stress in FD and its interplay with inflammation.

Oxidative stress has also been linked to the pathogenesis of other LSDs. For instance, in PD, patient-derived iPSC cardiomyocytes exhibited increased levels of oxidized glutathione (GSSG), suggestive of oxidative stress. A reduced mitochondrial β-oxidation was also reported, indicative of mitochondrial dysfunction [70]. In Niemann-Pick type C (NP-C) disease, an LSD characterized by the lysosomal accumulation of cholesterol and glycosphingolipids [71], patient-derived iPSCs showed an increased ROS production, accompanied by a downregulation of catalase, a key antioxidant enzyme [72], and a decrease in SOD activity [73]. A different study on primary fibroblasts from GD patients revealed disturbed mitochondrial membrane potential and elevated ROS production [74]. Together, these findings suggest the significance of oxidative stress not just in FD but also in other LSDs. A thorough understanding of the underlying mechanisms and impacts is crucial for developing effective therapeutic interventions for these complex diseases.

## Inflammation in Fabry Disease

### Immune Responses and Cardiac Implication

**Fig. 1:**
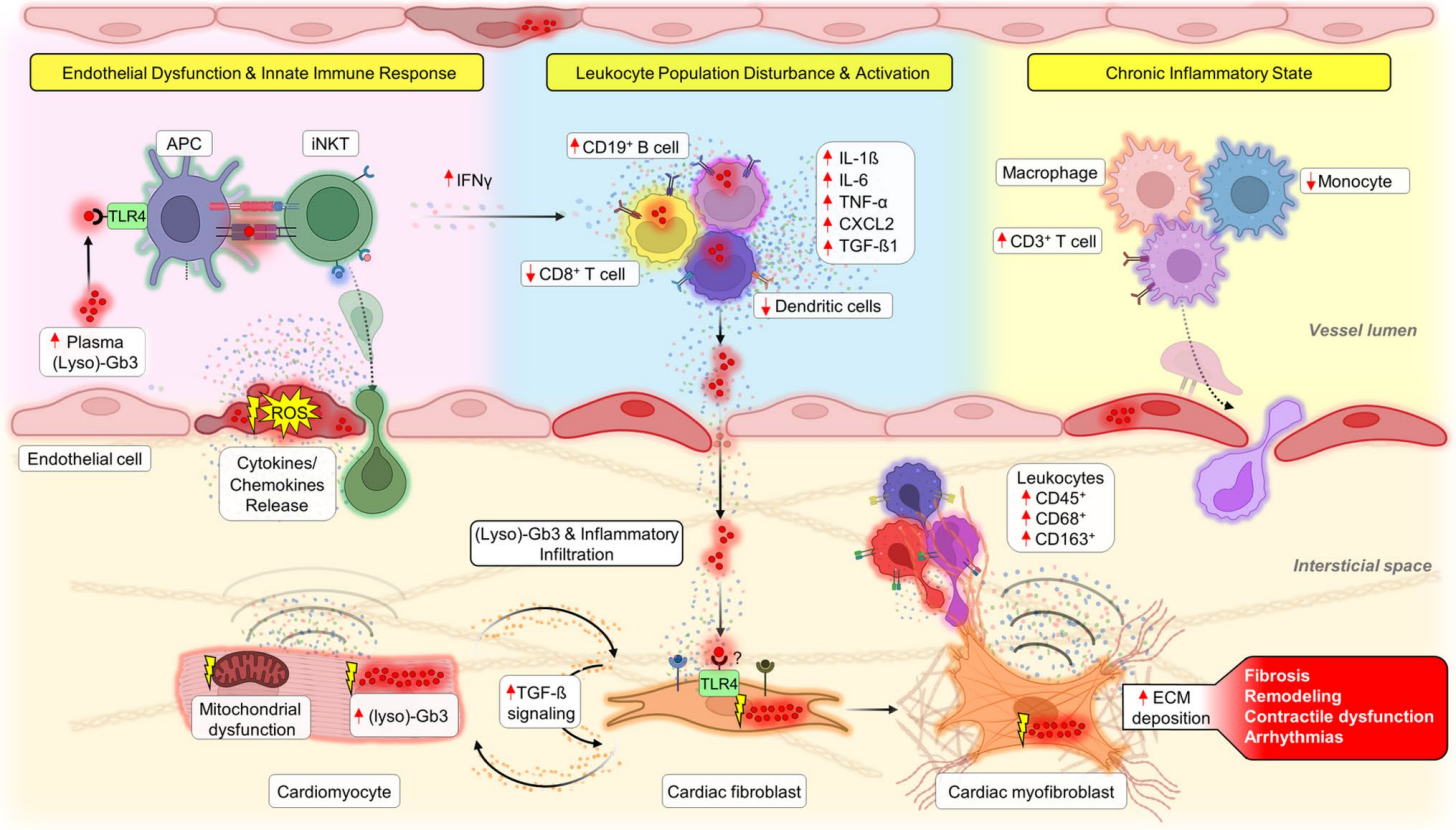
Integrated molecular pathways and cardiac implications in Fabry disease. Plasma globotriaosylceramide (Gb3) and globotriaosylsphingosine (lyso-Gb3), acting as both antigens and/or damage-associated molecular patterns (DAMPs) [34], bind to toll-like receptor 4 (TLR4) present on antigen-presenting cells (APCs). Upon this interaction, APCs enable the presentation of (lyso)-Gb3 to invariant natural killer T (iNKT) cells, thereby initiating pro-inflammatory signaling pathways, particularly leading to the release of interferon-gamma (IFN-γ; Pink field) [33, 99]. This interaction triggers a surge in pro-inflammatory mediators, predominantly released by peripheral blood mononuclear cells (PBMCs; blue field) [101•]. Concurrently, the shear stress and oxidative stress exerted on endothelial cells due to (lyso)-Gb3 deposition intensify local inflammatory cues, prompting iNKT cells to infiltrate the interstitium via the endothelial barrier [238]. This cascade amplifies cardiomyocyte stress, which is closely tied to mitochondrial dysfunction and (lyso)-Gb3 accumulation (orange field) [60•]. In this evolving inflammatory environment, macrophages, CD3^+^ T cells, and monocytes become prone to activation. These cells subsequently migrate and permeate the interstitium, further substantiating the chronic inflammatory response (yellow field) [27•]. Within the interstitial domain, (lyso)-Gb3 can also influence cardiac fibroblasts, promoting their activation. Herein, transforming growth factor-β (TGF-β) plays a critical role, driving myofibroblast differentiation and subsequent collagen deposition, accelerating the fibrotic progression and cardiac remodeling inherent in Fabry disease (orange field) [23–26, 27•]

Inflammation is an acute physiological process primarily orchestrated by the immune system in response to different stimuli, including damage-associated molecular patterns (DAMPs) and antigens (Fig. 1) [75]. DAMPs, released from stressed or injured cells, bind to pattern recognition receptors on macrophages, lymphocytes, neutrophils, and dendritic cells [76]. These interactions trigger immune and inflammatory responses, characterized by cell activation, migration, and the subsequent release of pro-inflammatory mediators, which further amplify immune cell recruitment [77]. While this inflammatory cascade is essential for immune defense, it can transition into a chronic inflammatory state when the initiating stimuli persist, culminating in autoinflammatory damage and organ dysfunction [78].

Immune responses and inflammation have been consistently linked to the pathogenesis of various LSDs. For instance, elevated plasma levels of inflammatory biomarkers, including tumor necrosis factor (TNF), interleukin-6 (IL-6), interleukin 1β (IL-1β), monocyte chemoattractant protein-1 (MCP-1), and galectin-1, have been observed in FD patients [79, 80•]. These biomarkers correlate with increased levels of cardiac remodeling and HF markers such as matrix metalloproteases (MMPs), brain natriuretic peptides, and mid-regional pro-atrial natriuretic peptides. A correlation was also established between these biomarkers and elevated plasma levels of lyso-Gb3 [80•]. Clinical profiling with echocardiogram and late gadolinium enhancement (LGE) imaging demonstrated myocardial LVH and cardiac fibrosis in these patients, reinforcing the positive correlation with plasma biomarkers. Notably, a cohort of FD patients with chronic kidney disease showed significantly higher levels of inflammatory biomarkers, suggesting an association with disease severity [80•].

Similarly, cytokines such as IL-1β, IL-6, TNF-α, and others like soluble IL-2 receptor, IL-1 receptor antagonist, and hepatocyte growth factor (HGF) are elevated in the plasma of GD patients and associated with disease severity [81, 82]. Chemokines like IL-8, macrophage inflammatory protein-1 (MIP-1), and MCP-1 are also reported to play pivotal roles in GD’s pathophysiology [83–85]. Chitotriosidase, an enzyme secreted by activated macrophages, has been reported to be elevated in numerous LSDs [86]. Chitotriosidase plasma levels and activity correlate with disease severity and serve as an indicator of therapeutic efficacy in GD [87]. Notably, increased plasma levels of chitotriosidase are documented in male FD patients, emphasizing its potential as a marker for lipid-laden macrophage accumulation [88]. Cytokines including IL-6, IL-8, and IL-10 are also elevated in NP-C patients [89]. These findings highlight the complex interplay of inflammatory and immune responses in LSDs, suggesting that chronic inflammation might be a shared underlying pathological mechanism in these diseases, opening avenues for potential therapeutic interventions.

Frustaci et al. [27•] highlighted the inflammatory nature of FD by reporting myocarditis in myocardial biopsies from FD patients, evidenced by increased CD3(+) T lymphocytes. This was associated with cell necrosis and increased plasma levels of anti-heart and anti-myosin autoantibodies, suggesting that autoimmunity may contribute to the progression of FD. Additionally, Hayashi et al. [90] identified elevated levels of lymphocytes and macrophage-related markers (i.e., CD45, CD68, and CD163) in myocardial biopsies from FD patients, confirming the infiltration of immune cells in the hearts of patients with Fabry cardiomyopathy (Fig. 1). These observations were accompanied by increased troponin I plasma levels and clinical symptoms like dyspnea, angina, arrhythmias, and cardiac dysfunction. Collectively, these observations reinforce the evolving characterization of FD as a chronic inflammatory disease.

Leukocyte population disturbances have been reported in FD patients, evidenced by an increase in CD19(+) cells and a reduction in monocytes, CD8(+) cells, and myeloid dendritic cells [91]. Altered expressions of the major histocompatibility complex (MHC) class II and CD1d were also observed in the monocytes of these patients [91]. Notably, similar alterations are observed in monocytes from GD patients [92]. It is hypothesized that these changes may influence antigen presentation and T cell responses [93, 94]. Studies in FD mice have shown a reduction in invariant natural killer T (iNKT) cells, with FD patients displaying alterations in iNKT cell subsets [67, 95]. Observations in GD mouse models and patients also revealed an increase in glucosylsphingosine-specific type II NKT cells, a subset with potential roles in disease pathology [96]. Similar anomalies in leukocyte populations have been observed in patients with mucopolysaccharidoses (MPSs), an LSD resulting from specific enzyme deficiencies [97]. Specifically, MPS type VI patients display decreased levels of NK cells and monocytes, accompanied by altered balance between naïve and memory T cells [98]. These leukocyte population disturbances across LSDs suggest potential implications for disease progression, which will be further explored in subsequent sections.

### Cellular and Molecular Mechanisms of Inflammation in Fabry Disease

Gb3 and lyso-Gb3 act both as an antigen and as a DAMP. When recognized by TLR4 on CD1d-expressing antigen-presenting cells, (lyso)-Gb3 can be presented to iNKTs [33, 99]. Antigen-recognized (lyso)-Gb3 in turn can trigger an immune response associated with the transcription of pro-inflammatory cytokines such as interferon-gamma (IFN-γ) [100]. (Lyso)-Gb3 can also trigger a pro-inflammatory response in peripheral blood mononuclear cells from FD patients, evidenced by the increased production of IL-1β, IL-6, and TNF-α (Fig. 1) [101•]. Notably, inhibiting TLR4 with a blocking antibody abolished these responses, suggesting a role in the local and systemic inflammatory state observed in FD patients [101•]. TLR4 activation can also contribute to inflammation by activating nicotinamide adenine dinucleotide phosphate (NADPH) oxidases, which subsequently increases ROS production [102]. This results in the nuclear translocation of the nuclear factor-kappa B (NF-κB), and the transcription of genes involved in immune response and inflammation [103, 104].

Insights from other LSDs have underscored the role of TLR4 activation in mediating inflammation and influencing disease pathogenesis. Specifically, abnormal glucosylceramide accumulation in GD macrophages triggers the TLR4-MyD88 pathway [105]. This activation leads to increased secretion of B cell chemoattractant C-X-C motif chemokine ligand 13 (CXCL-13), which is implicated in enhanced B cell trafficking. This process potentially contributes to tissue damage in affected organs. Additionally, fibroblasts from NP-C patients demonstrate elevated secretion of pro-inflammatory cytokines, including interferon-β, IL-6, and IL-8. This increased cytokine production was linked to endosomal accumulation of TLR4 [106]. The involvement of TLR4 in these conditions underscores its potential role in activating inflammatory pathways in various LSDs, thereby reinforcing its significance in FD.

The association between TLR4 activation and chronic inflammation observed in FD patients parallels findings in hypertensive heart diseases and chronic kidney disease [107, 108]. Notably, FD patients exhibit a significant prevalence of hypertension [109, 110], which has been attributed to autonomic dysfunction affecting cardiovascular structures [111, 112]. In these pathological settings, the hyperactivation of the renin-angiotensin system (RAS) emerges as a common pathogenic denominator, associated with chronic inflammation and organ damage [113]. The activation of RAS initiates with the secretion of renin, which converts angiotensinogen to angiotensin I. Subsequently, the angiotensin-converting enzyme (ACE) catalyzes the conversion of angiotensin I into angiotensin II (Ang-II), a potent vasoactive peptide and the primary RAS effector [114]. By binding to Ang-II receptor type 1 (AT1R), Ang-II triggers a pro-inflammatory cascade in different cell types, including immune, endothelial, and smooth muscle cells, which contribute to leukocyte recruitment, and the production of pro-inflammatory cytokines, contributing to endothelial dysfunction, cardiac remodeling, and vascular complications [115, 116].

In FD patients, sphingolipid accumulation is thought to reduce arterial compliance, consequently triggering a localized upregulation of the RAS. This process plays a significant role in the pathophysiology of FD, contributing to vascular complications [26]. Moreover, FD patients exhibit elevated plasma levels of renin, positively correlating with lyso-Gb3 plasma levels [117]. Given the identified association between TLR4 and NLR family pyrin domain–containing 3 (NLRP3) inflammasome, a critical mediator of inflammatory responses [118, 119], we hypothesize that this interplay might also play a role in the pathogenesis of FD. Supporting this idea, Corredor et al. [120] recently demonstrated that Ang-II acts synergistically with AT1R and TLR4 to activate the NLRP3 inflammasome in rat cardiac fibroblasts, resulting in myofibroblast differentiation and IL-1β secretion. This interplay operates through a Ca^2+^-dependent pathway involving phospholipase C and inositol 1,4,5-trisphosphate receptor. Although the specifics of this mechanism remain unclear, it is proposed to contribute to the inflammatory and fibrotic responses observed in various heart diseases [121, 122]. However, the implications of this RAS/TLR4/NLRP3 axis for FD remain unexplored, highlighting the need for more research.

Pro-inflammatory cytokines, reported to be elevated in FD patients, can amplify the inflammatory response by acting in a paracrine manner, thereby facilitating intercellular communication (Fig. 1). For instance, they can further stimulate cytokine release from cardiomyocytes [123], endothelial cells [124], and cardiac fibroblasts [125]. Of particular interest is the multifunctional cytokine transforming growth factor-β1 (TGF-β1), found elevated in plasma from FD patients and associated with fibrotic progression and cardiac remodeling [126]. This will be explored further in the subsequent section. A deeper understanding of the interplay between inflammation and the manifestations associated with FD is crucial for unraveling the complex mechanisms of this condition and improving patient care.

## Fibrosis in Fabry Disease

### The Role of Fibrosis in Cardiac Involvement

Fibrosis is a prominent feature in FD, characterized by an excessive accumulation of ECM components. This accumulation leads to the structural remodeling of affected organs [127]. In FD, cardiac fibrosis predominantly manifests as replacement fibrosis, characterized by collagen deposition following cardiomyocyte death, which is linked to abnormal Gb3 accumulation in these cells [128]. This fibrotic response primarily targets the basal posterolateral segments of the heart and extends transmurally as the disease progresses [129, 130]. The consequential fibrotic remodeling alters not just the morphology of the heart, but also its electrical properties, predisposing FD patients to arrhythmias and HF [20•, 131]. FD patients show a positive correlation between the extent of cardiac fibrosis and both ventricular arrhythmias and LV wall thickness, and this is associated with wall motion abnormalities, primarily in the fibrotic areas [132, 133, 134•]. Cardiac fibrosis in FD patients has been also associated with the severity of ventricular hypertrophy [20•, 128]. Notably, recent evidence shows that diastolic dysfunction is not a precursor for the onset of cardiac fibrosis, as this can be observed even in the absence of evident functional impairments [132].

Numerous studies have shown a correlation between elevated serum levels of collagen metabolism biomarkers and both the LV mass and occurrences of malignant ventricular arrhythmias (VA) in patients with Fabry cardiomyopathy [20•, 133]. Notably, increased levels of serum procollagen type I carboxyterminal pro-peptide (PICP), a marker of collagen synthesis, were observed at early preclinical stages in FD patients, even in the absence of cardiac dysfunction [133]. The suppression of MMPs, crucial enzymes for collagen degradation, may further exacerbate myocardial collagen deposition in these patients [133]. These observations suggest that monitoring such biomarkers could provide valuable insights into the disease’s progression, potentially aiding in a more nuanced assessment of Fabry disease’s cardiac impact.

Research has shown that the effectiveness of ERT in treating FD cardiac involvements is influenced by the severity of the fibrotic response. In particular, FD patients without replacement fibrosis experienced a decrease in LV mass and enhanced cardiac function after 3 years of ERT [129]. In contrast, patients with mild and severe fibrosis only observed a mild reduction in septal wall thickness, with no improvements in cardiac function, the extent of fibrosis, or serum collagen biomarkers [129]. Notably, patients with evident fibrosis had a higher incidence of VA, some of which led to sudden cardiac deaths [20•]. This association between cardiac fibrosis and severe cardiac events emphasizes the critical clinical implications of myocardial fibrosis in FD, emphasizing the necessity for early detection and more targeted therapeutic strategies.

Cardiac fibrosis in FD exhibits sex-dependent variations. In females, fibrosis can manifest at early disease stages, even in the absence of pronounced LVH. This contrasts with male patients, where LVH often precedes fibrosis [134•]. This sex-specific pattern might be influenced by X-chromosome inactivation impacting GLA gene expression. Specifically, one of the female X-chromosomes undergoes transcriptional silencing, leading to a mosaic expression pattern for X-linked genes, including the GLA gene [134•, 135, 136]. This mosaicism could lead to a partial compensation of the mutant cell defect, potentially influencing the onset of specific cardiac manifestations. This is a mechanism observed in female patients with other X-linked metabolic disorders [137]. In contrast, male patients, who possess only one X-chromosome, display a consistent expression of the mutated GLA gene across all cells [136]. The absence of a compensatory allele in males might exacerbate sphingolipid accumulation, providing to some extent a mechanistic explanation for the differences in the course of the disease and severity of symptoms between sexes [138–141]. However, several studies have not established a clear correlation between skewed X-chromosome inactivation and disease severity, indicating a need for further research [141–143]. The cardiac implications and sex-specific variations in FD presentation highlight the need for continued research. A comprehensive understanding is vital to enhance patient outcomes.

### Cellular and Molecular Mechanisms of Fibrosis

Central to fibrosis is the transforming growth factor-β1 (TGF-β1), a pro-fibrotic cytokine involved in the activation of fibroblasts and the trans-differentiation of myofibroblasts, responsible for ECM deposition [144]. Elevated plasma levels of TGF-β1 have been consistently observed in patients with various LSDs, including FD [82, 126, 145, 146]. Circulating TGF-β1 can originate from a variety of cells, including immune cells and fibroblasts, particularly in response to cellular stress or injury [147]. TGF-β1 interacts with TGF-β receptor type I (TGFBR1) and type II (TGFBR2) expressed in several cell types [148]. This interaction triggers both canonical and/or noncanonical signaling pathways. In the canonical pathway, TGFBR1 leads to the phosphorylation of SMAD family member 2/3 (SMAD2/3), promoting its interaction with SMAD4 and subsequent transcriptional regulation. TGF-β1 can also activate mitogen-activated protein kinase/Jun N-terminal kinase/p38 signaling (MAPK/JNK/p38) via a noncanonical pathway, in turn stimulating cell activation and trans-differentiation, and pro-fibrotic gene expression [149, 150].

In FD, elevated plasma levels of TGF-β1 are closely associated with cardiac hypertrophy and fibrosis (Fig. 1) [126]. Similarly, myocardial biopsies from MPS type I patients revealed hyperactivated TGF-β signaling, evidenced by an upregulation of phosphorylated SMAD2/3. These findings correlated with cardiac hypertrophy, stenosis in the coronary arteries, and fibrosis [151]. In MPS types VI and VII, enhanced production of TGF-β1 has also been observed, and this was accompanied by altered expression of MMPs [149]. An upregulation of TGF-β1 was also reported in mesenchymal stromal cells from GD type 1 patients, accompanied by an altered inflammatory secretome, contributing to immune disease manifestations [152]. These findings collectively highlight the detrimental effects of dysregulated TGF-β signaling and suggest its potential as a common fibrotic mechanism in various LSDs.

Sanchez-Niño et al. [153] proposed a direct role for lyso-Gb3 in promoting TGF-β1 production and trans-differentiation of renal cells into myofibroblasts. Using cultured human podocytes, the authors reported an increased expression of ECM proteins, including fibronectin I and collagen type IV, upon lyso-Gb3 treatment. Rozenfeld et al. [154] corroborated this association by showing elevated levels of TGF-β1 and α-smooth muscle actin (α-SMA), a marker of activated fibrogenic cells, in kidney biopsies from FD patients. These findings were accompanied by interstitial fibrosis and local inflammation markers, suggesting that lyso-Gb3 could be a key direct trigger for fibrotic responses, potentially influencing not only renal cells but also cardiac cells (Fig. 1). Lyso-Gb3 can also activate vascular endothelial cells, resulting in TGF-β1 secretion and thereby amplifying fibrosis [155]. In contrast, Choi et al. [156] found that lyso-Gb3 could inhibit proliferation, differentiation, and collagen synthesis in mouse adventitial fibroblasts through a process involving the downregulation of the Ca^2+^-activated potassium channel (KCa3.1). This contrasting evidence suggests that lyso-Gb3’s impact varies across different cell types, potentially associated with differences in the cellular microenvironment, receptor/channel expression, signaling pathways, or intracellular mechanisms.

Cardiac fibrosis in FD might also be linked to hemodynamic-independent RAS hyperactivation. Elevated serum levels of renin, which correlate with plasma lyso-Gb3 levels in FD patients [117], can lead to increased Ang-II production. Ang-II, in turn, can stimulate the activation of cardiac fibroblasts, leading to enhanced collagen deposition and α-SMA production, as well as TGF-β1 secretion [157–159]. Importantly, Ang-II-mediated ROS generation and the activation of the redox-sensitive transcription factor NF-κB play a role in this process [157, 160]. Ang-II can also promote the recruitment and transformation of bone marrow–derived fibroblast precursors, specifically CD34^+^ and CD45^+^, into myofibroblasts [161, 162]. This process is attributed to Ang-II-induced elevations in the chemokine stromal-derived factor (SDF)-1α levels in the heart [163]. Additionally, these precursor cells, together with endothelial cells, can undergo endothelial-to-mesenchymal transition and express α-SMA, potentially contributing to myocardial fibrosis [163, 164]. The interplay of TGF-β signaling, RAS activation, and inflammatory signaling in FD highlights the complex nature of cardiac fibrosis. Comprehensive research is essential to unravel these interconnected mechanisms and their impact on cardiac cells, paving the way for the development of targeted and effective therapeutic strategies.

## Innovative Therapeutic Strategies in Fabry Disease

### Mitochondrial-Targeted Interventions

Mitochondrial dysfunction in FD often manifests as a decline in mitochondrial respiratory chain function, bioenergetic deficits, and overproduction of ROS, which collectively exacerbate cellular dysfunction and promote disease progression [41]. Targeting these mitochondrial defects offers promising potential for therapeutic intervention in the management of the disease.

For example, coenzyme Q10 (CoQ10) treatment, involved in both ROS scavenging and mitochondrial ATP production [165], has been reported to alleviate mitochondrial dysfunction and reduce ROS production in Gaucher macrophages and NP-C patient-derived fibroblasts [166, 167]. Idebenone, a synthetic analog of CoQ10, has garnered attention in mitochondrial therapeutics for its dual capacity to act as an antioxidant and to facilitate electron transfer directly to complex III of the mitochondrial respiratory chain, bypassing complex I deficiencies [168]. This mechanism could prove especially beneficial for addressing mitochondrial disturbances associated with defects in respiratory chain enzymes, as reported in FD [48], and other LSDs [74, 169, 170].

EPI-743, another potent CoQ10 analog developed to treat inherited mitochondrial diseases, has also shown promising results in improving mitochondrial function and cellular energy metabolism [171, 172], by targeting key oxidoreductase enzymes such as NAD(P)H: quinone oxidoreductase 1. This, in turn, restores glutathione levels, commonly found altered across LSDs [63, 70, 173, 174], and improves mitochondrial redox status. Elamipretide, also referred as SS-31, is a mitochondrial-targeted tetrapeptide that has shown therapeutic promise in restoring mitochondrial function and bioenergetics in diverse clinical settings, including cardiomyopathies [175, 176], inflammatory diseases [177, 178], and mitochondrial disorders [179–181]. Elamipretide binds to and stabilizes cardiolipin, a phospholipid crucial for the optimal assembly and functioning of the mitochondrial respiratory chain. Consequently, this interaction leads to a reduction in mitochondrial ROS production, enhancement of oxidative phosphorylation, and augmentation of ATP production [182, 183].

Furthermore, the supplementation of thiamine (vitamin B1), serving as a cofactor for both the mitochondrial pyruvate dehydrogenase complex and TCA cycle enzymes [184], presents a promising approach warranting further exploration. The ability of thiamine to stimulate mitochondrial oxidative phosphorylation and enhance ATP production efficiency [185, 186] holds particular significance given the high glycolytic rates, and diminished oxidative phosphorylation observed in cells from FD mice and patients [52•, 187, 188]. These insights highlight the attractive potential of therapies targeting mitochondrial functions, paving the way for improved standard care in FD through the rectification of key metabolic dysfunctions.

### Antioxidant Strategies

Oxidative stress is increasingly recognized as pivotal in the pathogenesis of LSDs, including FD. This stress can trigger inflammatory responses and fibrosis, both correlating with disease progression and organ damage [189]. Research indicates that interventions targeting oxidative stress mitigation could offer promising therapeutic avenues for managing these diseases. Antioxidants such as N-acetylcysteine (NAC) are known to normalize pro-inflammatory cytokine production in NP-C fibroblasts [190], potentially through restoring trafficking and mitigating oxidative stress. The antioxidant GSH has demonstrated effectiveness in attenuating oxidative stress in renal FD in vitro models [66], whereas ascorbate supplementation has been found to decrease cerebral hyperperfusion in FD patients undergoing ERT [191]. The effect of ERT can be enhanced with the adjunct use of antioxidants like vitamin E, and ticlopidine, suggesting that a multi-pronged approach may be beneficial for FD patients [192–194].

Nanoparticles with antioxidant properties, such as polyethylene glycol–capped ceria-zirconia, have demonstrated their ability to reduce glycolipid accumulation, attenuate oxidative stress, and reduce pro-fibrotic cytokine production in FD patient-derived epithelial cells and podocytes, suggesting a potential role for such nanotechnologies in FD treatment [195]. Similarly, β-cyclodextrin nanoparticles have shown efficacy in reducing cholesterol accumulation and mitigating mitochondrial oxidative stress [167]. This highlights the therapeutic potential of nanoparticles combined with antioxidant therapy, offering a promising strategy for more comprehensive and effective management of LSDs, including FD, by addressing the interplay of metabolic dysfunction, oxidative stress, and inflammation inherent to the disease.

## Nutritional Strategies in Fabry Disease Management

### Ketogenic Diet

The ketogenic diet (KD), known for increasing ketone bodies like beta-hydroxybutyrate and acetoacetate, may serve as a potentially valuable nutritional adjunct for patients with FD and other LSDs. Studies have linked KD to lower serum levels of oxidative stress markers like malondialdehyde (MDA) and 8-OHdG [196]. Furthermore, an immunomodulatory role for KD and ketone supplementation, particularly beta-hydroxybutyrate, has been suggested. This is evidenced by the reduction of serum levels of pro-inflammatory cytokines and chemokines in both patients and animal models with chronic inflammation, correlating with cardiovascular benefits [197–201].

Various mechanisms have been proposed for the beneficial effects of KD, including the modulation of inflammatory signaling pathways such as NF-κB [202], NLRP3 inflammasome [203, 204], and the mitogen-activated protein kinase/extracellular signal-regulated kinase (MAPK/ERK) pathway [205], all of which have been reported to be disturbed in FD [206, 207], and other LDSs [208, 209]. Additionally, KD has been associated with improved mitochondrial function and ATP production [210, 211]. However, prolonged KD in some studies has been linked to reduced mitochondrial biogenesis and several health concerns, including cardiac complications and metabolic disturbances [212, 213], emphasizing the need for more comprehensive research and careful application in long-term dietary interventions.

### Acetyl-dl-Leucine Supplementation

Acetyl-dl-leucine, a derivative of the branched-chain amino acid leucine, has demonstrated therapeutic benefits in various LSDs [214]. Pre-symptomatic administration in animal models of NP-C disease and GM2 gangliosidosis (Sandhoff disease) not only delayed disease progression but also extended lifespan, suggesting a modulatory role in disease progression [214]. Specifically, acetyl-dl-leucine was shown to upregulate the pyruvate dehydrogenase E1-alpha subunit, which is associated with an increased conversion of pyruvate to acetyl-CoA, while downregulating pyruvate dehydrogenase kinase 2, the enzyme responsible for its inactivation [214]. This leads to an increased influx of acetyl-CoA into the TCA cycle, enhancing mitochondrial oxidative phosphorylation and ATP production [215].

Clinical studies have also highlighted the effectiveness of acetyl-dl-leucine, especially its l-enantiomer, in alleviating different symptoms in NP-C and GM2 gangliosidosis patients [214, 216]. Furthermore, the combination of acetyl-dl-leucine with substrate reduction therapy has shown synergistic effects, improving therapeutic outcomes in both animal models and NP-C patients [214]. These findings underscore the promising potential of acetyl-dl-leucine as a versatile adjunct in the treatment of LSDs, warranting exploration in FD.

### Omega-3 Fatty Acid Supplementation

Nutritional intervention involving omega-3 fatty acids, such as eicosapentaenoic acid (EPA), docosahexaenoic acid (DHA), and alpha-linolenic acid (ALA), has been shown to confer beneficial effects for clinical conditions associated with chronic inflammation and fibrosis [217, 218]. In human studies, omega-3 supplementation has been associated with lower serum biomarkers of inflammation and cardiac fibrosis in patients with ischemic HF and those who have suffered acute myocardial infarction [219, 220]. These studies also reported an association between diminished LV remodeling and improved systolic function, indicating potential clinical benefits for cardiovascular health. Notably, recent research has revealed that DHA and ALA can inhibit the KCa3.1 channel [221], which is involved in cardiac remodeling [222] and fibrosis [161]. This approach offers therapeutic possibilities in different LSDs, where disturbances in KCa3.1 channel function have been commonly observed. In FD, KCa3.1 functional alterations impact fibroblast and endothelial cells, and are associated with fibrosis and endothelial dysfunction [156, 223]. In NP-C and GD, altered KCa3.1 function is linked to immune responses and inflammation [156, 223–225]. However, the mechanisms underlying this inhibition and their clinical efficacy require further investigation.

### Flavonoid Intake

Emerging research suggests the therapeutic potential of flavonoid intake in managing inflammatory and fibrotic diseases, primarily through the inhibition of MyD88/NF-κB, NLRP3 inflammasome [226, 227], and the MAPK/ERK signaling pathways [228]. Total flavonoid intake inversely correlates with plasma inflammatory markers such as serum C-reactive protein and circulating inflammatory cytokines [229, 230]. Studies in animal models of HF have also highlighted the antifibrotic properties of flavonoid supplementation, particularly in reducing cardiac fibrosis, thereby improving cardiac function [231–233]. This effect is achieved by modulating the TGF-β/SMAD signaling pathway and activating silent information regulators 1 and 5 [231–233]. In vitro studies also show that flavonoids can attenuate Ang-II-induced cardiac fibroblast activation and ECM production, indicating a potential role in modulating RAS-associated fibrogenic responses [232, 234].

Quercetin, a dietary flavonoid, has been extensively studied for its cardiovascular benefits [235]. It effectively attenuates cardiac hypertrophy and fibrosis in various mouse models of HF, enhancing mitochondrial energy metabolism, dynamics, and biosynthesis [233, 236]. Research in mitochondria isolated from rat brains and hearts indicates that flavonoids, including quercetin, reduce mitochondrial ROS production by targeting key respiratory chain components, specifically complex I and the cytochrome c-cardiolipin complex [237]. These mechanisms hold promise for addressing mitochondrial and energetic disturbances associated with FD. Additionally, flavonoid-rich green tea, used alongside ERT, has demonstrated effectiveness in reducing oxidative stress in FD patients, evidenced by reduced levels of oxidative stress markers like MDA and heme-oxygenase-1 [194]. Collectively, these studies highlight the multifaceted role of flavonoids in modulating cellular and metabolic pathways, which could play a synergistic part in managing inflammation, oxidative stress, and associated complications in FD.

## Conclusions

FD, characterized by GLA gene mutations, results in deficient α-Gal A enzyme activity, leading to systemic accumulation of glycosphingolipids, primarily Gb3, and lyso-Gb3. This accumulation significantly impacts cardiovascular systems and manifests with a broad clinical spectrum influenced by age, sex, genetic variations, and enzyme activity levels. However, FD pathogenesis extends beyond glycosphingolipid accumulation, encompassing mitochondrial dysfunction, characterized by imbalances in energy molecules like PCr, ADP, and ATP, and alterations in mitochondrial structure and respiratory function. These alterations are closely linked with oxidative stress, where an imbalance between ROS production and antioxidant defense systems exacerbates tissue damage, driving the activation of pro-inflammatory and pro-fibrotic signaling pathways. A comprehensive understanding of these disturbances is necessary for addressing the progression and treatment challenges in FD, especially concerning cardiac involvement.

Inflammation in FD is driven by dysregulated immune responses, playing a critical role in the disease’s pathogenesis. The activation of innate immune system components, including the TLR-4 signaling pathway, is associated with an increased release of pro-inflammatory cytokines, contributing to both local and systemic chronic inflammation. The hyperactivation of the RAS is also implicated in establishing a low-grade inflammation and redox imbalance that characterizes FD patients, which further exacerbates cardiac and vascular complications. Inflammation, coupled with mitochondrial function defects and oxidative stress, may significantly contribute to the pathogenesis of Fabry cardiomyopathy, characterized by left ventricular hypertrophy and myocardial fibrosis that predispose to cardiac arrhythmias and HF.

The development of LVH in FD patients, often a consequence of the aforementioned pathological processes, is a critical factor in the progression and severity of cardiac manifestations. Cardiac fibrosis, recognized as a pivotal factor in FD progression, is influenced by pro-inflammatory and pro-fibrotic cytokines, especially through the TGF-β/SMAD signaling pathway and interconnected pathways including NF-κB, MAPK/ERK, and RAS. The presence and severity of fibrosis are critical determinants of the response to therapies such as ERT and represent a critical juncture in FD, beyond which current treatments become less effective, underscoring the urgent need for innovative therapeutic approaches.

Future research should focus on targeting these molecular disruptions, potentially by exploring the integration of targeting mitochondrial functions and antioxidant therapies with existing treatments. Personalized medicine, tailored to individual disease profiles, may also exert beneficial roles in patient care for FD. Further research into nutritional strategies is also encouraged. This includes exploring the effects of KD, omega-3 fatty acid supplementation, acetyl-dl-leucine, and flavonoid intake. These dietary approaches might have the potential to provide supportive care by addressing metabolic and oxidative stress challenges and modulating inflammatory and fibrotic responses, thereby offering a more holistic approach to managing FD.
